# Longitudinal 18F-Fluorodeoxyglucose Positron Emission Tomography (18F-FDG PET) Findings in Korsakoff Syndrome After Rapid Weight Loss During Tirzepatide Therapy: A Case Report

**DOI:** 10.7759/cureus.110101

**Published:** 2026-06-02

**Authors:** Viviane T Crelier, Alexandra S Vidal, Nathane B Rezende, Marcos R Figueiredo, Sergio A de Almeida, Jose K Jorge, Gabriel R de Freitas

**Affiliations:** 1 Neurology, Fluminense Federal University, Rio de Janeiro, BRA; 2 Radiology, Rede D'Or, Rio de Janeiro, BRA; 3 Cardiology, Rede D'Or, Rio de Janeiro, BRA; 4 Neurology, D'Or Institute for Research and Education (IDOR), Rede D'Or, Rio de Janeiro, BRA

**Keywords:** alcohol use disorder, fdg pet, glp-1 receptor agonists, korsakoff syndrome, rapid weight loss, thiamine deficiency, tirzepatide, wernicke encephalopathy

## Abstract

We report a case of a 50-year-old man with obesity and chronic alcohol use disorder who developed Korsakoff syndrome in the setting of rapid weight loss temporally associated with tirzepatide therapy, a dual glucagon-like peptide-1/glucose-dependent insulinotropic polypeptide (GLP-1/GIP) receptor agonist. Within weeks of treatment initiation, he presented with profound memory impairment and anosognosia. Initial brain magnetic resonance imaging (MRI) was unremarkable, but 18F-fluorodeoxyglucose positron emission tomography (18F-FDG PET) revealed hypometabolism in the mammillary bodies with progressive involvement of the thalamus and brainstem. Despite intravenous thiamine replacement, cognitive deficits persisted. This case raises concern that rapid weight loss and reduced nutritional intake during GLP-1-based therapy may contribute to the development of Wernicke-Korsakoff syndrome in vulnerable individuals, particularly those with chronic alcohol use disorder, and underscores the importance of nutritional assessment and consideration of thiamine supplementation when initiating these treatments.

## Introduction

Glucagon-like peptide-1 (GLP-1) receptor agonists have transformed the management of type 2 diabetes mellitus and obesity by improving glycemic control and promoting substantial weight loss. More recently, dual agonists targeting both GLP-1 and glucose-dependent insulinotropic polypeptide (GIP) receptors, such as tirzepatide, have demonstrated even greater efficacy in weight reduction [1]. In addition to their metabolic benefits, these agents are being investigated for potential neuroprotective effects [2] and as possible treatments for conditions such as alcohol use disorder [3,4].

Despite their therapeutic benefits, the use of GLP-1-based therapies requires careful nutritional assessment, particularly in vulnerable populations. Rapid and significant weight loss may precipitate critical micronutrient deficiencies, most notably thiamine deficiency, through reduced oral intake, depletion of nutritional reserves, and increased metabolic demand during catabolic states. Thiamine plays a crucial role in neuronal metabolism, and its deficiency is a well-established risk factor for Wernicke-Korsakoff syndrome (WKS), a severe neuropsychiatric disorder. Wernicke encephalopathy classically presents with the triad of confusion, gait ataxia, and oculomotor abnormalities, although the full triad is often absent [5]. Individuals with alcohol use disorder are particularly susceptible to thiamine depletion and may therefore be at increased risk of WKS when exposed to abrupt nutritional compromise.

Importantly, some patients may not present with the classic triad of Wernicke encephalopathy and instead develop prominent anterograde amnesia and confabulation, a clinical presentation characteristic of Korsakoff syndrome (KS), the chronic amnestic phase of the thiamine-deficiency spectrum [6]. Early recognition of this atypical presentation is essential, as the acute phase may be subclinical or missed entirely. Although early thiamine administration may reverse Wernicke encephalopathy, progression to KS is often associated with persistent cognitive deficits.

Neuroimaging can support the diagnosis of WKS, particularly in cases with atypical or incomplete clinical presentations. Magnetic resonance imaging (MRI) may reveal signal abnormalities in the thalami, mammillary bodies, and periaqueductal gray matter, although these findings are not always present. In addition, 18F-fluorodeoxyglucose positron emission tomography (18F-FDG PET) can provide functional information by detecting regional metabolic abnormalities that may precede or persist beyond structural changes seen on MRI [6].

Although this biological rationale is clinically plausible, evidence linking GLP-1-based therapies to WKS remains limited and largely indirect, particularly in the presence of established risk factors such as chronic alcohol use disorder. Rapid weight loss and reduced nutritional intake may represent contributing factors in susceptible individuals rather than a direct causal mechanism.

Here, we report a case of KS occurring in temporal association with rapid weight loss after tirzepatide initiation in a patient with chronic alcohol use disorder.

## Case presentation

Clinical history and initial evaluation

A 50-year-old man with a history of obesity (body mass index 30 kg/m²) and long-standing alcohol use disorder presented to the emergency department with a one-week history of severe memory impairment and lack of awareness of his deficits (anosognosia). Collateral history obtained from family members revealed several years of chronic heavy alcohol consumption, with an estimated daily intake of approximately 460 g of ethanol, without recent isolated escalation. Prior to symptom onset, he was fully independent in daily activities and had no known history of cognitive impairment or functional decline.

The clinical timeline is summarized as follows: Six weeks prior to admission, he initiated a weight-loss regimen that included anastrozole, tamoxifen, and a testosterone implant. Two weeks later, tirzepatide was introduced. Over the subsequent four weeks, he experienced a rapid 15 kg weight loss (approximately 10% of body weight), associated with markedly reduced food intake. During the same period, family members also reported persistence of his chronic heavy alcohol consumption, with possible worsening nutritional compromise. Progressive behavioral changes and prominent memory deficits developed during the week preceding admission, leading to hospitalization.

During the period of rapid weight loss, family members reported marked appetite suppression after tirzepatide initiation, with significantly reduced oral intake. No persistent nausea or vomiting was reported. Importantly, at presentation, the patient did not exhibit the classic triad of Wernicke encephalopathy, as there were no gait abnormalities, ophthalmoplegia, or nystagmus, and the predominant manifestation was severe amnestic syndrome with anosognosia.

Neurological examination revealed no gait abnormalities, nystagmus, or oculomotor dysfunction. Cognitive screening using the Montreal Cognitive Assessment (MoCA) yielded a score of 23/30, with deficits in delayed recall (0/5), verbal fluency, and temporal orientation.

Given the presence of multiple potential contributing factors, including chronic alcohol use disorder, rapid weight loss, reduced oral intake, and concurrent hormonal therapy, the case was approached as a multifactorial nutritional and neuropsychiatric syndrome.

Autoimmune and infectious investigations were unremarkable. Testing was negative for antinuclear antibodies, rheumatoid factor, antineutrophil cytoplasmic antibodies (both cytoplasmic and perinuclear patterns), and anti-Ro and anti-La antibodies. Cerebrospinal fluid polymerase chain reaction testing was negative for herpes simplex virus types 1 and 2, varicella-zoster virus, cytomegalovirus, Epstein-Barr virus, and enteroviruses. Serologic testing for human immunodeficiency virus and syphilis was also negative. The initial MRI was normal.

Serum thiamine levels at admission were 118.9 nmol/L (reference >70 nmol/L). However, serum thiamine may not accurately reflect intracellular thiamine status, particularly in the setting of recent supplementation. More reliable markers of tissue deficiency include whole-blood thiamine diphosphate and erythrocyte transketolase activity, which were not available in this case. Importantly, the patient had started oral vitamin supplementation five days prior to hospitalization, including thiamine (100 mg), pyridoxine (100 mg), and cyanocobalamin (5000 mcg), which may have masked a preceding deficiency. Laboratory findings at admission are summarized in Table 1.

**Table 1:** Laboratory findings at admission TSH, thyroid-stimulating hormone; free T4, free thyroxine; AST/TGO, aspartate aminotransferase; ALT/TGP, alanine aminotransferase; GGT, gamma-glutamyl transferase

Parameter	Patient Value	Reference Range	Unit
Serum thiamine (vitamin B1)	118.9	>70	nmol/L
Sodium	139	135-145	mmol/L
TSH	1.5	0.4-4.0	mIU/L
Free T4	1.1	0.8-1.8	ng/dL
AST (TGO)	56	10-40	U/L
ALT (TGP)	42	7-56	U/L
GGT	82	9-48	U/L

Given the clinical presentation of subacute severe anterograde amnesia, anosognosia, recent rapid weight loss, and chronic alcohol use disorder, a diagnosis of KS was established. High-dose intravenous thiamine therapy was initiated (500 mg every eight hours for three days, followed by 300 mg daily for seven days). Despite treatment, there was no significant improvement in cognitive function during hospitalization. The patient was discharged after seven days with oral thiamine supplementation (300 mg daily for 60 days).

18F-FDG PET imaging findings

As the patient developed a persistent disproportionate amnestic syndrome despite an initially unrevealing structural MRI, FDG PET was performed to evaluate cerebral glucose metabolism and to investigate functional involvement of memory-related diencephalic and limbic networks. The examination was not intended to replace MRI or thiamine-based clinical diagnosis, but to provide metabolic characterization of an otherwise structurally subtle syndrome and to help assess alternative causes of persistent cognitive decline.

On the initial FDG PET scan, increased metabolic activity was observed in the bilateral thalami (right: +2.0 SD; left: +1.7 SD) (Figures 1A, 1B) and brainstem (+2.5 SD) (Figures 1E, 1F), suggestive of early compensatory or reactive hypermetabolism. In contrast, the mammillary bodies demonstrated reduced FDG uptake (Figures 1E, 1F), consistent with regional dysfunction.

**Figure 1:**
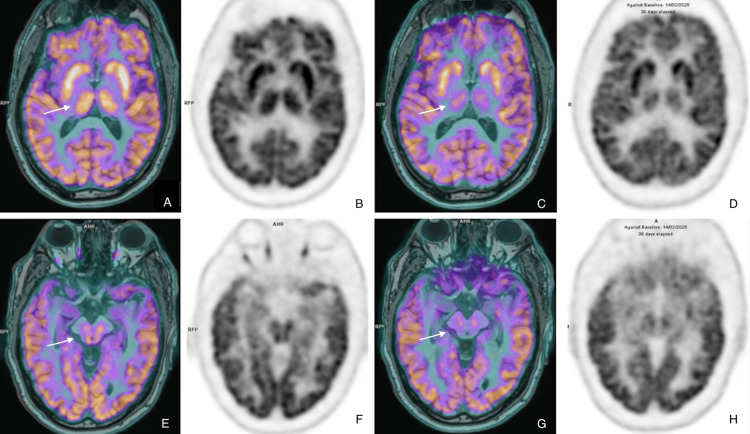
Serial 18F-FDG PET/MRI fusion and PET-only images demonstrating a biphasic metabolic trajectory in the thalamic and brainstem regions (A, B) Initial scan at the thalamic level demonstrating increased 18F-FDG uptake in the bilateral thalami (right: +2.0 SD; left: +1.7 SD), consistent with early hypermetabolism. White arrows highlight the thalamic regions of interest. (C, D) Follow-up scan performed on day 39 at the same anatomical level, showing reduced uptake in the bilateral thalami (right: -1.1 SD; left: -2.3 SD), indicating progression to hypometabolism. White arrows indicate the same thalamic regions on follow-up imaging. (E, F) Initial scan at the brainstem level demonstrating increased 18F-FDG uptake (+2.5 SD), suggestive of early metabolic stress. White arrows highlight the brainstem region of interest. (G, H) Follow-up scan at the same level showing reduced brainstem uptake (+1.7 SD), consistent with subsequent metabolic decline. White arrows indicate the corresponding brainstem region on follow-up imaging. Quantitative values are expressed as SD differences relative to an age-matched reference database, with positive values indicating relative hypermetabolism and negative values indicating relative hypometabolism. These findings illustrate a dynamic biphasic pattern, with early hypermetabolism followed by later hypometabolism in the same anatomical regions. 18F-FDG PET, 18F-fluorodeoxyglucose positron emission tomography; MRI, magnetic resonance imaging

A follow-up FDG PET scan performed 39 days later showed a marked decline in thalamic and brainstem metabolism. Brainstem uptake decreased to +1.7 SD, while thalamic activity fell below normal limits (right thalamus -1.1 SD; left thalamus -2.3 SD). Hypometabolism in the mammillary bodies persisted (Figures 1C, 1D, and Figures 1G, 1H).

This evolving pattern, with initial thalamic and brainstem hypermetabolism followed by subsequent hypometabolism, was interpreted as consistent with progressive metabolic decompensation affecting diencephalic and brainstem structures typically involved in WKS.

Follow-up

At a follow-up visit two months later, the patient’s family reported slight improvement in memory, although anosognosia persisted. Repeat MoCA testing showed a score of 24/30, with persistent memory deficits.

Functionally, the patient remained partially dependent for instrumental activities of daily living, particularly medication management, financial tasks, and appointment scheduling. Functional assessment using the Pfeffer Functional Activities Questionnaire yielded a score of 12 (cutoff ≥9 indicating functional impairment) [7].

During follow-up, oral intake had improved, and the patient had achieved partial nutritional recovery, with stabilization of weight after the initial rapid loss. Family members also reported a reduction in alcohol consumption compared with the period immediately preceding hospitalization, although chronic alcohol use disorder remained an ongoing clinical concern.

Neuropsychological testing with the Free and Cued Selective Reminding Test (FCSRT) demonstrated severe episodic memory impairment, characterized by low free recall, limited benefit from cueing, and a high number of intrusions (n=17) [8-10]. Immediate recall was relatively preserved, suggesting intact encoding but marked impairment in consolidation and retrieval, consistent with an amnestic syndrome.

Brain MRI performed three months after symptom onset revealed a subtle linear hyperintensity on T2-weighted and fluid-attenuated inversion recovery (FLAIR) sequences in the dorsomedial region of both thalami (Figure 2).

**Figure 2:**
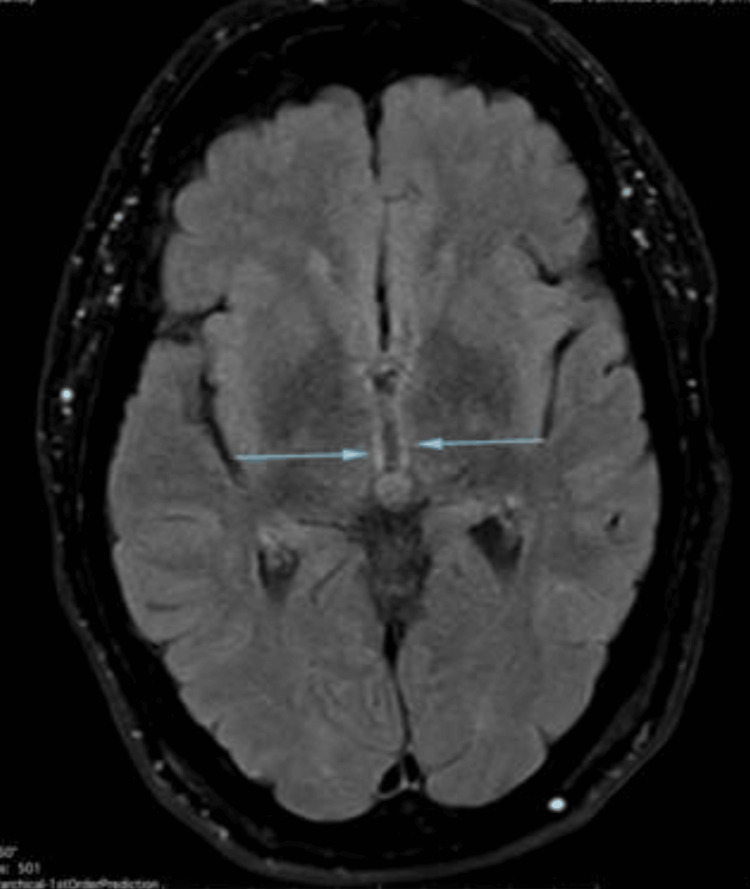
Brain MRI performed three months after symptom onset, showing subtle linear hyperintensity on FLAIR sequences in the dorsomedial region of both thalami FLAIR, fluid-attenuated inversion recovery; MRI, magnetic resonance imaging

The patient, a professional engineer and business owner, was unable to return to work because of persistent cognitive and executive dysfunction.

## Discussion

This case illustrates a clinically important and likely multifactorial presentation of KS occurring in temporal association with rapid weight loss during GLP-1/GIP receptor agonist therapy in a patient with chronic alcohol use disorder. Chronic alcohol use disorder was likely the major predisposing factor for thiamine depletion, while rapid weight loss and reduced nutritional intake may have acted as additional precipitating factors. Despite normal serum thiamine levels and unremarkable initial MRI findings, the clinical presentation and FDG PET imaging supported the diagnosis of KS. Notably, FDG PET revealed a dynamic biphasic metabolic pattern characterized by early hypermetabolism in the thalami and brainstem, followed by subsequent hypometabolism in the same regions on follow-up imaging.

Multiple conditions may precipitate WKS through thiamine deficiency, including chronic alcohol use, persistent vomiting, malabsorption, bariatric surgery, and severe dietary restriction. Thiamine stores are limited and may become critically depleted within two to three weeks of sustained nutritional compromise [11]. In this context, rapid weight loss, such as that associated with GLP-1 receptor agonists, may further increase this risk, particularly in individuals with chronic alcohol use disorder. Without adequate thiamine supplementation, severe neurologic complications, including WKS, may occur.

Alternative diagnoses were considered during the diagnostic workup. Alcohol-related dementia was considered less likely given the acute-to-subacute onset and the prominent amnestic syndrome with temporal association to rapid nutritional compromise. Hepatic encephalopathy was not supported by the clinical presentation or laboratory evaluation. Autoimmune and infectious encephalitides were also considered unlikely in light of the negative cerebrospinal fluid and serologic studies, as well as the characteristic neuroimaging and neuropsychological findings.

GLP-1 receptor agonists, such as semaglutide and liraglutide, promote appetite suppression, delayed gastric emptying, and substantial weight loss, reaching up to 16% in clinical trials with semaglutide. Tirzepatide, a dual GLP-1/GIP receptor agonist, has demonstrated even greater efficacy, with weight reductions approaching 20.9% [12]. These agents are increasingly prescribed for obesity and metabolic disease and are also being explored for potential neurologic applications. Interestingly, semaglutide has been associated with reductions in alcohol craving and consumption, suggesting potential therapeutic roles in alcohol use disorder [13]. However, in susceptible individuals, rapid weight loss and reduced oral intake during GLP-1-based therapy may contribute to nutritional compromise and increase the risk of thiamine-deficiency syndromes.

Neuroimaging can support the diagnosis of WKS, particularly in cases with atypical or incomplete clinical presentations. MRI commonly demonstrates symmetrical T2/FLAIR hyperintensities in the medial thalami, mammillary bodies, tectal plate, and periaqueductal gray matter in approximately 50%-80% of cases [14]. FDG PET findings in WKS typically include hypometabolism in diencephalic structures, particularly the thalamus and mammillary bodies, as well as in the medial temporal lobes, posterior cingulate cortex, and occasionally the frontal cortex [15-17]. These metabolic abnormalities likely reflect disruption of the diencephalic-limbic memory network underlying the cognitive deficits characteristic of WKS.

In the present case, FDG PET demonstrated a dynamic biphasic metabolic trajectory. Initial hypermetabolism in the thalami and brainstem was followed by progressive metabolic decline in these regions on follow-up imaging. This pattern resembles findings observed in experimental models of thiamine deficiency. Murata et al. [18] reported similar metabolic changes in thiamine-deprived rat brain slices, with early increases in glucose uptake followed by irreversible metabolic decline after thiamine repletion. To our knowledge, this may represent the first clinical description of a comparable metabolic trajectory in a human case of KS.

Although these findings provide valuable functional insight and may have contributed to diagnostic confidence in the setting of an initially unremarkable MRI, they should be interpreted cautiously. The metabolic pattern observed in this case may not be specific and should not be generalized beyond the context of a single case report. Rather than establishing FDG PET as a biomarker, this case suggests that it may offer complementary functional information in selected atypical presentations.

This report has several limitations. The patient’s prior use of multivitamin supplementation may have partially masked an underlying thiamine deficiency at the time of testing, potentially underestimating the severity of micronutrient depletion. Additionally, although FDG PET provided valuable functional insight in this case, its limited availability and high cost may restrict its routine clinical use. Further studies are needed to clarify the potential role of PET imaging in the early detection and longitudinal assessment of WKS.

Importantly, this case underscores the need for careful nutritional assessment in patients undergoing pharmacologic weight loss therapy, particularly those with alcohol use disorder. Screening for nutritional risk factors and early thiamine supplementation may help prevent severe neurologic complications associated with rapid weight loss.

## Conclusions

This case highlights a serious and likely multifactorial neurologic complication occurring in temporal association with rapid weight loss during GLP-1/GIP receptor agonist therapy in a patient with chronic alcohol use disorder. In this context, chronic alcohol use disorder likely represented the major predisposing factor for thiamine depletion, while the potent anorexigenic effects and associated nutritional compromise may have acted as additional contributing factors in a vulnerable individual. Notably, this report describes a dynamic biphasic FDG PET pattern in WKS, characterized by initial thalamic and brainstem hypermetabolism followed by subsequent hypometabolism, paralleling findings from experimental models of thiamine deficiency. Although these findings provide valuable functional insight, they should be interpreted cautiously and may offer complementary information in selected atypical cases, particularly when MRI findings are initially unremarkable.

Given the expanding use of GLP-1-based therapies, clinicians should carefully assess alcohol use, psychiatric comorbidities, and nutritional status before initiating treatment. Early recognition of nutritional risk and consideration of thiamine supplementation may help reduce the risk of severe neurologic complications in high-risk patients.
